# Real-World Efficacy and Safety of Avelumab Plus Axitinib in Metastatic Renal Cell Carcinoma: Results from the Ambispective RAVE-Renal Study

**DOI:** 10.3390/curroncol32010011

**Published:** 2024-12-27

**Authors:** Ilya Tsimafeyeu, Vyacheslav Chubenko, Olga Baklanova, Alexey Kalpinskiy, Sufia Safina, Andrei Lebedinets, Vladislav Petkau, Elvira Parsadanova, Maria Turganova, Aleksei Shkurat, Natalia Tovbik, Elena Tkacheva, Yulia Anzhiganova, Olga Novikova, Varvara Bragina, Ruslan Zukov, Rashida Orlova

**Affiliations:** 1 Bureau for Cancer Research—BUCARE, Moscow Office, 125047 Moscow, Russia; 2 Saint-Petersburg Clinical Scientific and Practical Center for Specialized Types of Medical Care Oncological, 197110 Saint-Petersburg, Russia; 3 Irkutsk Regional Cancer Center, 664048 Irkutsk, Russia; 4 P.A. Hertsen Moscow Oncology Research Center—National Medical Research Center of Radiology, 125284 Moscow, Russia; 5 Republican Dispensary of Tatarstan, 420029 Kazan, Russia; 6 SBHI Leningrad Regional Clinical Hospital, 194291 Saint-Petersburg, Russiashkurat.aleksei@yandex.ru (A.S.); 7 Sverdlovskiy Regional Oncological Dispensary, 620036 Ekaterinburg, Russia; 8 Sakhalin Regional Cancer Center, 693010 Yuzhno-Sakhalinsk, Russia; 9 Novosibirsk Regional Cancer Center, 630108 Novosibirsk, Russia; 10 Amursky Regional Cancer Center, 675006 Blagoveshchensk, Russia; 11 Primorsky Regional Cancer Center, 690091 Vladivostok, Russia; 12 A.I. Kryzhanovsky Krasnoyarsk Regional Cancer Center, 660133 Krasnoyarsk, Russia; 13 Khabarovsk Regional Cancer Center, 680042 Khabarovsk, Russia; 14 Tver Regional Cancer Center, 170100 Tver, Russia; 15 V.F. Voyno-Yasenetsky Krasnoyarsk State Medical University, 660022 Krasnoyarsk, Russia; 16 Saint-Petersburg State University, 199034 Saint-Petersburg, Russia

**Keywords:** avelumab plus axitinib, first-line therapy, metastatic renal cell carcinoma, RAVE-Renal ambispective study

## Abstract

Background: The RAVE-Renal study was conducted to evaluate the real-world efficacy and safety of avelumab plus axitinib as a first-line therapy for patients with metastatic renal cell carcinoma (mRCC). Methods: RAVE-Renal was a multicenter, noninterventional, ambispective study with both retrospective and prospective components. The study included adult patients with histologically confirmed mRCC, measurable disease per RECIST version 1.1, and no prior systemic therapy. Patients received avelumab (800 mg intravenously every 2 weeks) plus axitinib (5 mg orally twice daily). The primary endpoints were median progression-free survival (PFS) and objective response rate (ORR). The secondary endpoints included median OS, 1-year overall survival (OS) rate, and safety. Results: A total of 125 patients from 13 sites were enrolled, with a median follow-up of 16.1 months. The median age was 61.0 years. The study population comprised 35.3% favorable, 49% intermediate, and 15.7% poor IMDC risk patients. The median PFS was 14.9 months (95% CI, 11.72–19.08). The ORR was 44.3% (95% CI, 32.5–56.1). The clinical benefit rate was 93.4%. The 1-year OS rate was 71.2%, with the median OS not reached. Any-grade treatment-related adverse events (TRAEs) occurred in 99 (79.2%) cases, including grade ≥3 TRAEs in 24 (19.2%). Conclusions: Avelumab in combination with axitinib showed clinical benefits in a real-world setting, consistent with findings from a pivotal trial. The regimen was effective and well tolerated across various patient subgroups.

## 1. Introduction

The incidence of renal cell carcinoma (RCC) is growing worldwide, and along with it, the number of cases of metastatic RCC (mRCC) is increasing [1]. The advent of novel therapies has revolutionized the treatment landscape for this malignancy, offering new hope for improved patient outcomes. Immunotherapy, particularly when combined with targeted agents, harnesses the immune system to recognize and combat cancer cells, providing a novel approach to tackling mRCC [2].

The phase 3 JAVELIN Renal 101 trial has been a pivotal study in this context, demonstrating the potential benefits of combining avelumab, an anti-PD-L1 antibody, with axitinib, a tyrosine kinase inhibitor, as a first-line treatment for mRCC. This trial revealed that patients treated with the combination exhibited a significantly improved objective response rate (ORR) and progression-free survival (PFS) compared to those treated with the standard therapy, sunitinib [3,4]. These findings are particularly important as they suggest enhanced efficacy across various patient subgroups, including those with different numbers of International Metastatic Renal Cell Carcinoma Database Consortium (IMDC) risk factors and varying numbers of tumor sites. The combination of avelumab and axitinib has shown generally favorable outcomes not only in terms of ORR but also in the extent of tumor shrinkage achieved. These benefits were observed regardless of PD-L1 expression, highlighting the versatility of this treatment regimen. The combination was approved in the United States in 2019 and has since been used around the world [5].

In light of these promising clinical trial results, there is a growing interest in evaluating the real-world effectiveness of avelumab and axitinib as a first-line therapy for mRCC. Our RAVE-Renal study aims to bridge the gap between controlled clinical trial environments and clinical practice, providing valuable insights into the real-world outcomes associated with avelumab and axitinib in untreated patients with metastatic renal cancer. By doing so, we seek to validate the clinical trial findings and further establish the role of immunotargeted therapy.

## 2. Materials and Methods

### 2.1. Study Design and Treatment

The RAVE-Renal study was a multicenter, noninterventional, ambispective study that included both prospective and retrospective components. In the retrospective part of this study, no more than 50% of patients were permitted, provided they met the eligibility criteria and initiated treatment no later than 6 months before the study commenced. The inclusion criteria for this study comprised histologically confirmed mRCC, patients aged 18 years or older at diagnosis; measurable disease according to the Response Evaluation Criteria in Solid Tumors (RECIST), version 1.1; and no history of systemic therapy. Patients with uncontrolled medical conditions (such as unstable angina pectoris, recent myocardial infarction, symptomatic congestive heart failure, or acquired or inherited bleeding disorders, or thrombosis), or those who had previously received adjuvant therapy for non-metastatic RCC were deemed ineligible for this trial. Additionally, patients involved in other clinical trials or treated with other systemic anticancer therapies were excluded. PD-L1 expression was not a mandatory inclusion criterion.

The patients were treated with avelumab at a flat dose of 800 mg intravenously every 2 weeks and axitinib at 5 mg orally twice daily. Dose reductions for avelumab were not permitted, while axitinib dose reductions to 3 mg or 2 mg, as well as dose increases to 7 mg or 10 mg twice daily, were allowed based on tolerance and clinical judgment. Treatment continued until disease progression, unacceptable toxicity, or other criteria for discontinuation occurred. The data were accessed for research purposes on 31 January 2023. The authors did not have access to information that could identify individual participants during or after data collection.

The RAVE-Renal study adhered to the principles outlined in the Declaration of Helsinki and received approval from the Principal Investigators and Study Group. All patients provided written informed consent to receive the immunotargeted therapy.

### 2.2. Endpoints and Assessments

The co-primary endpoints of the RAVE-Renal study were median PFS and ORR. Secondary endpoints included the median OS, 1-year overall survival (OS) rate, and safety.

Disease progression was assessed based on radiological and clinical data, with changes in therapy and death also serving as markers of disease progression. Follow-up evaluations were conducted every 8 (±2) weeks using computed tomography (CT) until confirmed disease progression, the date of death, or the last visit if the patient was alive. Response to treatment was measured according to the RECIST, version 1.1. Adverse events (AEs) were graded according to the National Cancer Institute Common Terminology Criteria for Adverse Events (NCI CTCAE), version 5.0.

This study also examined the switching from first-line therapy to subsequent-line therapies. Switching to the next line of treatment was defined as a change in therapy due to disease progression or toxicity. In some patients, immunohistochemical PD-L1 expression was evaluated in tumor samples using the SP263 clone (Ventana Medical Systems).

### 2.3. Statistical Analysis

The final efficacy and safety analyses included all patients who received at least one dose of avelumab and axitinib. Baseline patient characteristics and treatment regimens were summarized using descriptive statistics, including means, medians, and proportions. Quantitative data were expressed as mean ± standard deviation, and the Mann–Whitney U test, a nonparametric test, was employed to analyze variables that did not follow a normal distribution, assessing relationships between these variables.

Survival time was calculated from the start of treatment to either the day of death (for OS) or the day of disease progression or death from any cause (for PFS). The one-year survival rate was defined as the percentage of patients still alive 12 months after starting treatment. Survival curves were generated using the Kaplan–Meier method. Associations between outcomes and clinical or demographic factors were assessed using Kaplan–Meier analyses and log-rank tests.

All *p*-values reported were two-sided, with values less than 0.05 considered statistically significant. Analyses were conducted within a 95% confidence interval (CI). All statistical procedures were performed using IBM SPSS Statistics Base v22.0 software (SPSS, Inc., Chicago, IL, USA).

## 3. Results

### 3.1. Patient Characteristics

A total of 125 patients from 13 sites were recruited for this study, with a median follow-up of 16.1 months. Seventy (56%) patients were enrolled prospectively. Patient baseline characteristics are detailed in Table 1. At the initiation of treatment with avelumab plus axitinib, the median age of patients was 61.0 years (range, 37–74 years), with 31% of patients aged 65 years or older. Among the 102 patients with assessed IMDC risk, 35.3% were classified as being of favorable risk, while 49% were of intermediate risk, and 15.7% were of poor risk. The most common histological type was clear-cell carcinoma, observed in 98% of patients. A majority of patients (77%) had two or more metastatic sites including metastases in the lung (73%), lymph nodes (42%), bone (24.3%), liver (15.7%), and brain (3%). Additionally, 82% of patients had a history of prior nephrectomy. PD-L1 expression was assessed in 18 (14.4%) cases, with 11.1% testing positive.

### 3.2. Efficacy and Safety

**Figure 1:**
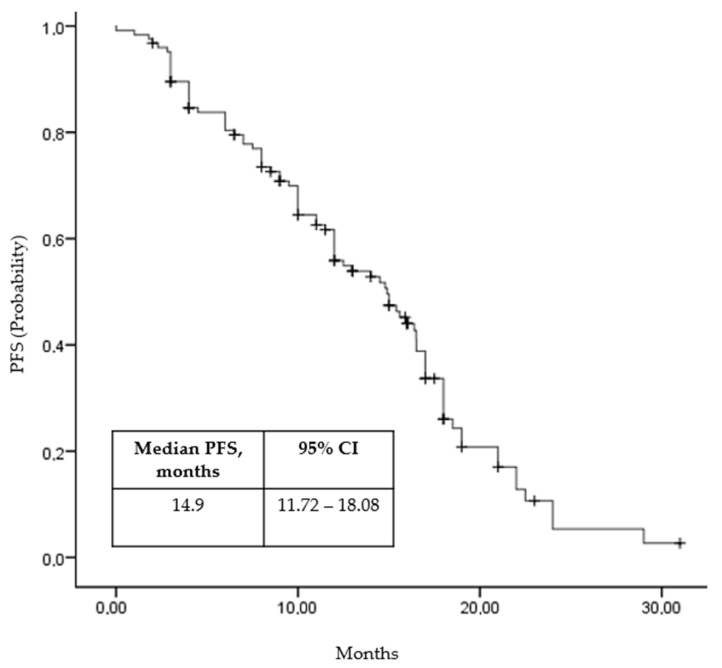
Progression-free survival (Kaplan–Maier curve).

The median PFS for the study population was 14.9 months (95% CI, 11.72 to 19.08 months; Figure 1). A univariate analysis revealed a significant difference in median PFS between patients with favorable and intermediate–poor IMDC risk, with favorable-risk patients experiencing a median PFS of 28.2 months compared to 10.0 months for those with intermediate–poor risk (*p* = 0.03). Additionally, patients with a history of nephrectomy had a better median PFS (16.0 months) compared to those without prior nephrectomy (5.1 months; *p* = 0.02). In patients with a time from diagnosis to treatment of metastatic disease of less than 1 year, the median PFS was numerically lower at 6.5 months compared to 17.6 months for those with a longer time interval; however, this difference did not reach statistical significance (*p* = 0.06). There was an observed trend suggesting that patients with a neutrophil-to-lymphocyte ratio (NLR) below the median had better PFS outcomes, with a median of 17.6 months compared to 5.1 months for those with NLR above the median (*p* = 0.04). The analysis did not identify significant differences in PFS based on other patient and treatment characteristics, including age (<65 vs. ≥65, *p* = 0.61), gender (male vs. female, *p* = 0.46), number of metastatic organs (1 vs. ≥2, *p* = 0.49), presence of liver (*p* = 0.65) and bone metastases (*p* = 0.16), and fibrinogen levels (below vs. above median, *p* = 0.3). A multivariate analysis confirmed these findings (Table 2).

**Figure 2:**
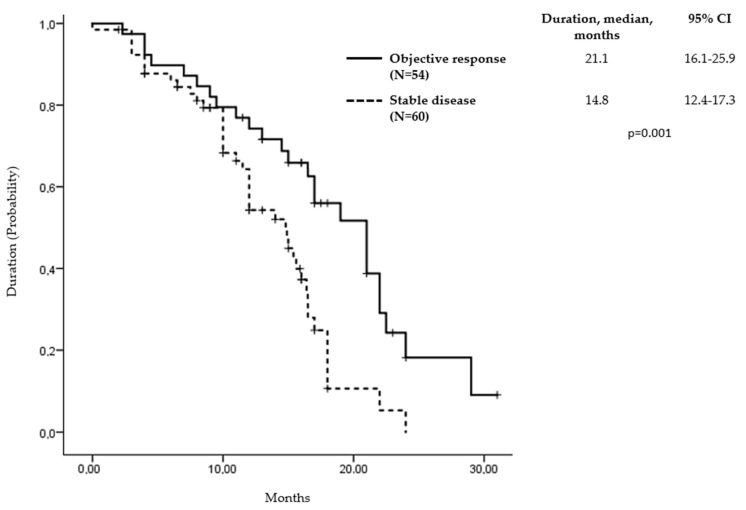
Duration of response and stable disease in assessed patients.

Response to avelumab and axitinib therapy was evaluated in 122 (97.6%) patients. The assessed ORR was 44.3% (95% CI, 32.5 to 56.1), with 3 patients (2.5%) achieving complete responses and 51 patients (41.8%) achieving partial responses. The responses were durable, with a median duration of 21.1 months (95% CI, 16.1 to 25.9; Figure 2). Three patients who achieved a complete response had clear cell histology, metastases to more than two organs, and a history of nephrectomy with development of metastatic disease within 1 year and were classified as intermediate risk. The median time to complete response was 4.7 months, with all responses ongoing. Additionally, 60 patients (49.1%) experienced stable disease, while 8 patients (6.6%) had progressive disease. The clinical benefit rate, defined as the proportion of patients who had a response or stable disease, was 93.4%.

As of the data cutoff, 50 patients (40%) were still receiving treatment under the trial. Among the patients who discontinued the study therapy due to disease progression, 32 out of 75 (43%) received a second-line therapy, including lenvatinib/everolimus, cabozantinib, everolimus, sunitinib, and nivolumab.

The median OS was not reached, with a 1-year OS rate of 71.2%. Patients who had not experienced disease progression by the time of the last follow-up exhibited a significantly longer OS compared to those with disease progression (median OS not reached vs. 18.7 months, *p* = 0.004).

In all treated patients, any-grade TRAEs occurred in 99 (79.2%) cases, including grade ≥3 TRAEs in 24 (19.2%). The most serious adverse events were immune colitis, arterial hypertension, and infusion-related reaction. High-dose glucocorticoids (≥1 mg/kg of prednisolone or equivalent) were administered to five patients (4.0%) to manage immune-related adverse events associated with avelumab plus axitinib.

## 4. Discussion

The RAVE-Renal ambispective study offers insights into the real-world application of avelumab plus axitinib as a first-line treatment for mRCC, demonstrating promising efficacy and safety outcomes. When compared to clinical trial settings, such as the JAVELIN Renal 101 trial [3,6], and other real-world studies, including those conducted in Japan (J-DART) [7] and the UK [8], several parallels and divergences emerge, underscoring the complexities of treating mRCC in diverse patient populations (Table 3).

The JAVELIN Renal 101 trial established the combination of avelumab and axitinib as a superior option compared to sunitinib, with a median PFS of 13.9 months and an ORR of 59.7% [3,6]. The RAVE-Renal study findings of a median PFS of 14.9 months and an ORR of 44.3% align closely with these results, suggesting consistent efficacy in both clinical trial and real-world settings. However, differences in patient demographics, inclusion criteria, and treatment protocols could account for the slight variations observed. For instance, the JAVELIN Renal 101 trial was a controlled, randomized study, while RAVE-Renal was an ambispective, non-interventional study, potentially introducing selection and reporting biases.

The J-DART study, a multicenter, retrospective, observational analysis of 48 patients, reported a median PFS of 15.3 months and an ORR of 48.8% [7]. These results are comparable to the RAVE-Renal study, albeit with a little higher ORR observed in the J-DART study. The J-DART study also reported a slightly higher complete response rate of 7.0% compared to 2.5% in RAVE-Renal. The J-DART cohort was older, with a median age of 69 years, and had a higher proportion of patients with intermediate and poor risk according to the IMDC criteria. Despite these differences, both studies highlight the effectiveness of avelumab and axitinib across varying patient profiles, reinforcing the robustness of this combination therapy.

The UK multicenter, retrospective trial included a cohort of 130 patients with a median age of 67.1 years, with a substantial proportion of patients being male (74%) with favorable IMDC risk (39%) [8]. This study observed a relatively lower prevalence of clear cell carcinoma at 88%, compared to the prevalence seen in RAVE-Renal and Javelin Renal 101, and a smaller proportion of patients had undergone prior nephrectomy, with only 68% having had the surgical procedure. A median PFS of 13.5 months closely aligned with the PFS observed in both the prospective and ambispective studies. Notably, the UK study reported an ORR of 62%, which is substantially higher than the ORR observed in RAVE-Renal. This discrepancy may be attributed to the longer follow-up period of 36 months in the UK study, allowing more time for patients to achieve partial or complete responses, as well as potentially capturing late-responders who may have been missed in shorter follow-up periods. The 1-year OS rate was 81.5% in the UK study, higher than that reported in the RAVE-Renal (71.2%). The adverse event profile in the UK study was consistent with known safety concerns associated with avelumab and axitinib, with diarrhea, fatigue, and oral mucositis being the most common.

Moreover, in the RAVE-Renal study, avelumab was administered at a fixed dose of 800 mg, differing from the weight-based dosing regimen (10 mg/kg) used in the JAVELIN Renal 101 trial. Nevertheless, this variation did not impact the safety outcomes. TRAEs were observed in the majority of patients, consistent with the known safety profile of avelumab plus axitinib. However, severe TRAEs (grade ≥ 3) occurred in only one fifth of patients and high-dose glucocorticoids were required in 4.0% of patients to manage immune-related adverse events. Despite the relatively high incidence of all grades of TRAEs, the rate of severe events and need for glucocorticoids remained within an acceptable range, emphasizing the importance of vigilant monitoring and prompt management in routine practice. These findings highlight the need for strategies to prevent and manage toxicities, ensuring the continued tolerability of this effective treatment combination.

The RAVE-Renal study design as a non-interventional, ambispective study presents several limitations. The lack of randomization and control group inherently limits the ability to draw definitive conclusions about the efficacy of avelumab plus axitinib compared to other therapies. The retrospective component, which included 44% of patients who initiated treatment up to six months before study commencement, could introduce recall bias and inconsistencies in data collection, particularly in assessing response and progression. Moreover, the absence of PD-L1 testing is another limitation, as PD-L1 expression could potentially stratify patients based on a likely response to the combination therapy. Furthermore, the median follow-up period of 16.1 months, while adequate for assessing ORR and PFS, may not fully capture OS outcomes and late-onset adverse events. Additionally, this study’s real-world nature, while providing valuable practical insights, also means that patient management could vary significantly across different sites, potentially influencing the outcomes [9].

## 5. Conclusions

In conclusion, the RAVE-Renal study underscores the efficacy and safety of avelumab plus axitinib in real-world practice, corroborating the findings from the JAVELIN Renal 101 trial and other real-world studies. While demonstrating substantial clinical benefits, this study also highlights the nuances and challenges of treating mRCC in diverse settings. The consistent findings across different trials reaffirm the role of the immunotargeted therapy as a viable first-line option for mRCC.

## Figures and Tables

**Table 1:** Patient characteristics.

Age (Years), Median (Range)	61.0 (37–74)
Gender, N (%)	
Male	92 (73.6)
Female	33 (26.4)
**Geographic region, N (%)**	
Europe	51 (41)
Asia	74 (59)
**IMDC risk, N (%)**	
Favorable	36 (35)
Intermediate	50 (49)
Poor	16 (16)
**Subtype of RCC, N (%)**	
Clear cell	123 (98)
Papillary	1 (1)
Collecting duct	1 (1)
**Site of metastases, N (%)**	
Lung	91 (73)
Lymph nodes	52 (42)
Bone	30 (24)
Liver	20 (16)
Adrenal	17 (14)
Brain	4 (3)
Other (soft tissues, pancreas, ovary, pleura, peritoneum)	9 (7)
**Organs with metastases, N (%)**	
1	29 (23)
≥2	96 (77)
**History of nephrectomy, N (%)**	
Yes	103 (82)
No	22 (18)
**Metastatic disease at the time of diagnosis, N (%)**	38 (30)
**Neutrophil-to-lymphocyte ratio, median (range)**	2.06 (0.5–7.4)
**Fibrinogen (baseline), g/L, median (range)**	4.3 (2.4–8.5)

**Table 2:** Multivariable model of factors associated with progression-free survival.

Factor	Odds Ratio (95% CI)
Age (<65 vs. ≥65)	0.69 (0.42–1.18)
Gender (male vs. female)	0.71 (0.47–1.33)
Number of metastatic organs (1 vs. ≥2)	0.85 (0.63–1.79)
Time from diagnosis to treatment of metastatic disease (≤1 vs. >1 year)	1.15 (0.37–3.16)
NLR (< median vs. ≥ median)	3.09 (1.75–6.28)
IMDC risk (favorable vs. intermediate–poor)	3.22 (1.93–5.9)
History of nephrectomy (yes vs. no)	4.94 (2.60–8.15)

CI, confidential interval; NLR, neutrophil-to-lymphocyte ratio; IMDC, International Metastatic Renal Cell Carcinoma Database Consortium.

**Table 3:** The key results from the RAVE-Renal, J-DART, UK real-world, and JAVELIN Renal 101 randomized studies.

Study	N (% of Favorable Risk)	Median PFS, Months	ORR, %	CR, %	1-Year OS Rate (%)	Median Response Duration, Months	Grade ≥ 3 AEs, %
RAVE-Renal	125 (35)	14.9	44.3	2.5	71.2	21.1	19.2
J-DART	48 (16.7)	15.3	48.8	7.0	-	-	-
UK Study	130 (39)	13.5	62	5.0	81.5	14.9	13.0
JAVELIN Renal 101	442 (19.3)	13.9	59.7	5.7	-	19.4	66.8

N, number of patients; PFS, progression-free survival; ORR, objective response rate; CR, complete response; OS, overall survival; AEs, adverse events.

## Data Availability

The datasets generated during and/or analyzed during the current study are available from the corresponding author on reasonable request.
